# Urea Concentration and Haemodialysis Dose

**DOI:** 10.5402/2013/341026

**Published:** 2012-11-01

**Authors:** Aarne Vartia

**Affiliations:** Dialysis Unit, Savonlinna Central Hospital, Keskussairaalantie 6, P.O. Box 111, 57101 Savonlinna, Finland

## Abstract

*Background*. Dialysis dose is commonly defined as a clearance scaled to some measure of body size, but the toxicity of uraemic solutes is probably associated more to their concentrations than to their clearance. *Methods*. 619 dialysis sessions of 35 patients were modified by computer simulations targeting a constant urea clearance or a constant urea concentration. *Results*. Urea generation rate *G* varied widely in dialysis patients, rather independently of body size. Dialysing to *eKt*/*V* 1.2 in an unselected patient population resulted in great variations in time-averaged concentration (TAC) and average predialysis concentration (PAC) of urea (5.9–40.2 and 8.6–55.8 mmol/L, resp.). Dialysing to equal clearance targets scaled to urea distribution volume resulted in higher concentrations in women. Dialysing to the mean HEMO-equivalent TAC or PAC (17.7 and 25.4 mmol/L) required extremely short or long treatment times in about half of the sessions. *Conclusions*. The relation between *G* and *V* varies greatly and seems to be different in women and men. Dialysing to a constant urea concentration may result in unexpected concentrations of other uraemic toxins and is not recommended, but high concentrations may justify increasing the dose despite adequate *eKt*/*V*, std EKR, or std *K*/*V*.

## 1. Introduction

The morbidity caused by uraemic toxins is probably associated more to their concentrations than to their clearances. Urea is a marker of dialysable uraemic toxins, which, however, are not produced and eliminated in a stoichiometric proportion to urea [1].

Big patients are supposed to need more dialysis than small: higher clearance (*K*) and/or more time (*t*). Equation

(1) $$\begin{array}{c} \frac{K*t}{V}=\ln\left(\frac{C_{0}}{C_{t}}\right), \end{array}$$

where *V* is urea distribution volume, ln natural logarithm, *C*_0_ predialysis, and *C*_*t*_ postdialysis concentration, describes the simplest urea kinetic model. It seems just what we want: a measure of dialysis dose automatically scaled to body size (represented by *V*) and calculated from only two simple variables. It is the basis of the popular second generation Daugirdas equation, which includes empiric correction factors for ultrafiltration and urea generation [2, 3]. 

The classic single-pool variable-volume urea kinetic model [4, 5] allows to take into account both ultrafiltration and urea generation individually. It requires iterative calculation and correct value of actual dialyser clearance (*K*_*d*_) to give correct urea generation rate *G* and distribution volume *V*. The double-pool model is even more accurate. 

*V* is a kinetic parameter required in modelling and simulating dialysis. But is *V*-scaled clearance the best variable to correlate dialysis dose to outcome? Small patients have worse outcome in haemodialysis than big if *Kt*/*V* is used as the dosing guideline [6–8]. An observational study based on a large material [9] and the prospective randomized HEMO trial [10] suggested that women—but not men—may benefit from higher urea reduction ratio (URR) or equilibrated *Kt*/*V* (*eKt*/*V*). 

It has been stated that dialysis intensity should be dimensioned to the metabolic needs instead of the size of the body [11]. Protein catabolic rate (PCR) or protein equivalent of nitrogen appearance (PNA) is a measure of protein metabolism [12]. Urea is a product of protein catabolism like probably many uraemic toxins. Urea generation rate *G* is used in the present report as the descriptor of protein metabolism. 

In the NCDS study [13, 14], higher time-averaged concentration (TAC) of urea was associated with worse outcome. Using a constant urea concentration as the target—like in the NCDS—may lead to a vicious cycle: the underdialysed patient loses his or her appetite → dietary protein intake (DPI), PCR, *G* and concentrations decrease → the dialysis dose will be diminished further. This can be avoided by using clearance instead of concentration as the dosing guide as presented by Gotch and Sargent [15] after the NCDS. Since then almost all studies and guidelines correlating outcome to dialysis dose have been based on *V*-scaled clearance (*Kt*/*V*) or fractional removal (URR). Only few defend concentration-based dosing [16–18]. 

Equivalent renal urea clearance (EKR, Casino and Lopez [19]) and std *K* (Gotch [20]) take the treatment frequency and residual renal function (RRF) into account and were intended to be used in comparing dialysis doses in different schedules and to continuous dialysis and renal function. EKR and std *K* are based on the definition of clearance (*K*):

(2) $$\begin{array}{c} K=\frac{E}{C}. \end{array}$$

In steady state, the excretion rate equals the generation rate: *E* = *G*, so

(3) $$\begin{array}{c} K=\frac{G}{C}. \end{array}$$

In EKR, *C* is the time-averaged concentration (TAC), in std *K*, the average predialysis concentration (peak average concentration, PAC). std *K* is scaled to body size by dividing by *V* and expressed as std *K*/*V*. Dividing EKR by *V* yields a variable called here as std EKR. *G*, *V*, TAC, and PAC can be determined by kinetic modelling.

(4) $$\begin{array}{c} \text{EKR}=\frac{G}{\text{TAC}}, \end{array}$$

(5) $$\begin{array}{c} \text{std}\;\text{EKR}=\frac{\text{EKR}}{V}\;=\frac{G}{\text{TAC}*V}, \end{array}$$

(6) $$\begin{array}{c} \text{std}\;K=\frac{G}{\text{PAC}}, \end{array}$$

(7) $$\begin{array}{c} \text{std}\;\;K/V=\frac{\text{std}\;K}{V}=\;\frac{G}{\text{PAC}*V}. \end{array}$$

The most practical unit of std EKR and std *K*/*V* is /wk. In std EKR, the term “std” means dividing EKR by *V* in (5); in std *K*/*V* it means dividing *G* by PAC in (6). 

With a constant clearance, *C* is linearly proportional to *G*. With a constant std EKR or std *K*/*V*, TAC and PAC are linearly proportional to *G*/*V*, a patient-specific variable not dependent on dialysis. 

Using a clearance scaled to *G* as the target means dialysing to a constant urea concentration:

(8) $$\begin{array}{c} \text{generation-scaled}\;\text{clearance}\;\;\frac{K}{G}=\frac{1}{C}. \end{array}$$

The required clearance *K* is determined by *G* and the desired concentration (3); *V* is needed as a kinetic parameter—not as a scaling factor—for creating the prescription (*K*_*d*_ and *t*_*d*_) in an intermittent schedule. 

The purpose of this study is to pay attention to the wide variation of concentrations when dialysing to a constant *eKt*/*V*, std EKR, or std *K*/*V* and to show with computer simulations what happens if we try to dialyse to a constant concentration. 

## 2. Subjects and Methods

### 2.1. Patients and Dialysis Sessions

619 consecutive urea kinetic modelling sessions of 35 unselected haemodialysis patients were included (Table 1). 

The dialysis dose prescription was not unambiguously defined. The patient's clinical condition, fluid removal requirement, and both *eKt*/*V* and single-pool std *K*/*V*, and predialysis urea concentration from the previous kinetic modelling sessions were taken into account. There were some long nightly, but not daily sessions. Dialysis time varied between 240 and 494 min, frequency between 1.75 and 4.45/wk, and *K*_*d*_ between 137 and 263 mL/min. The patients were encouraged by a dietician to use a diet containing protein 1.2 g/day/kg of normal weight (*V*/0.58). Protein intake was not actually measured.

### 2.2. Urea Kinetic Modelling

In the routine care of haemodialysis patients, a single-pool variable volume urea kinetic modelling with three blood samples and interdialysis urine collection was done once per month. Afterwards double-pool calculations were done by methods modified from the Solute-Solver programme [21] as described earlier [22]. Plasma concentrations were converted to plasma water concentrations before calculations and back to plasma concentrations in the results. Time-averaged concentration (TAC) and average predialysis concentration (PAC), needed in calculating EKR and std *K*, were determined after equalizing the schedule to a symmetric one as described earlier [23], using the double-pool model. TAC and PAC are expressed as external pool water concentrations converted to plasma concentrations. All clearances are expressed as plasma values. *V* is the sum of the external (*V*_*e*_) and internal (*V*_*i*_) pools at the end of the dialysis session.

### 2.3. Simulations

The dialysis sessions were modified by computer simulations with the double-pool UKM. After the patient-specific parameters *V*, *G*, renal urea clearance (*K*_*r*_), and fluid removal requirement have been determined by UKM, *K*_*d*_ and *t*_*d*_ can be varied in dialysis simulations and concentrations, *eKt*/*V*, std EKR and std *K*/*V* calculated. It is also possible to compute the required *K*_*d*_ or *t*_*d*_ to achieve a desired concentration or std EKR or std *K*/*V*.

## 3. Results

### 3.1. Urea Generation Rate *G* and Distribution Volume *V*

**Figure 1:**
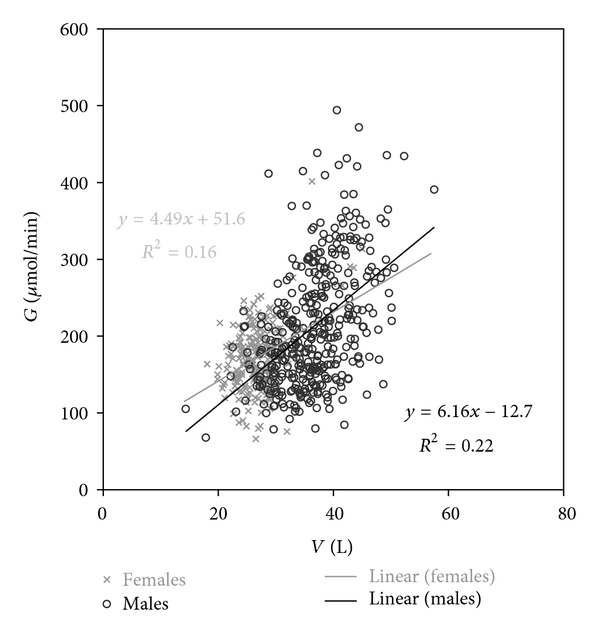
*G*  versus *V*.

*G* and *V* are true—not simulated—patient-specific variables independent of dialysis. The range of *G* in this material is 66–494 *µ*mol/min (variation 7.5-fold, Table 1) and *G*/*V* (*G* scaled to *V*) 2.0–14.3 *µ*mol/min/L (7.1-fold). The correlation between *G* and *V* is weak (Figure 1).

### 3.2. Determining HEMO-Equivalent std EKR, std *K*/*V*, TAC, and PAC with *K*_*r*_ 0 mL/min

In the HEMO study [24], the mean *eKt*/*V* in the standard dose group was 1.16 ± 0.08/session. In the present study, the material was “dialysed” (simulated) 3∗4 h/wk without residual renal function (RRF) to *eKt*/*V* 1.2 by adjusting *K*_*d*_ as described in detail earlier [22]. The mean *K*_*d*_ was 189 mL/min. The mean HEMO standard dose equivalent std EKR was 3.44/wk and std *K*/*V* 2.40/wk with little variation. Instead, TAC and PAC varied 6.8–6.5-fold (Table 2). The mean HEMO-equivalent TAC was 17.7 mmol/L (equal to the lower target in the NCDS) and PAC 25.4 mmol/L. 

std EKR and std *K*/*V* mean values corresponding to *eKt*/*V* 0.9 were 2.74/wk and 2.05/wk, respectively.

### 3.3. Dialysing to HEMO-Equivalent std EKR and std *K*/*V* with Actual *K*_*r*_

The material was dialysed (simulated) 3∗4 h/wk with actual *K*_*r*_ to the mean HEMO-equivalent std EKR and std *K*/*V* targets 3.44/wk and 2.40/wk by adjusting *K*_*d*_. 

**Figure 2:**
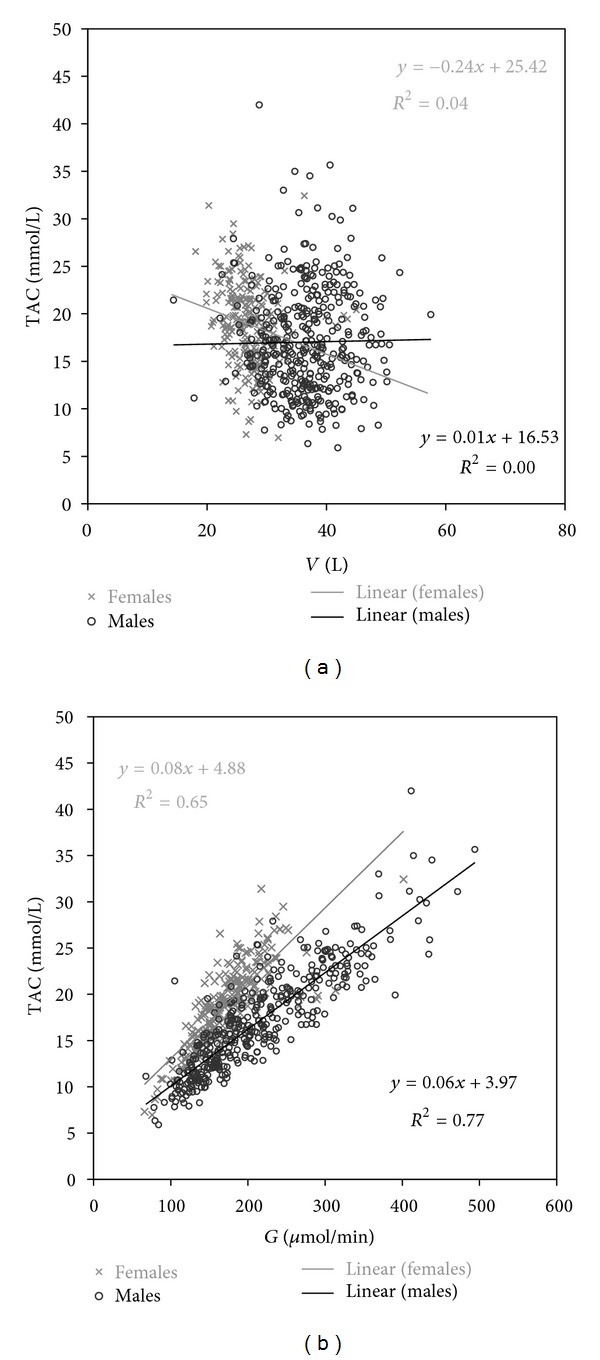
TAC versus *V* and *G* with std EKR 3.44/wk in a 3 × 4 h/wk schedule, *K*_*d*_ adjusted.

With std EKR target, TAC had a trend to be higher in women with lower *V*. With all levels of *G*, TAC was higher in women. The correlation between TAC and *V* is weak, between TAC and *G* quite strong (Figure 2). With std *K*/*V* target, the relations of PAC to *V* and *G* are similar (not shown). The mean TAC and PAC were higher in women than in men when dialysed to a constant *eKt*/*V*, std EKR, or std *K*/*V* (Table 3). 

### 3.4. Dialysing to HEMO-Equivalent TAC and PAC with Actual *K*_*r*_

**Figure 3:**
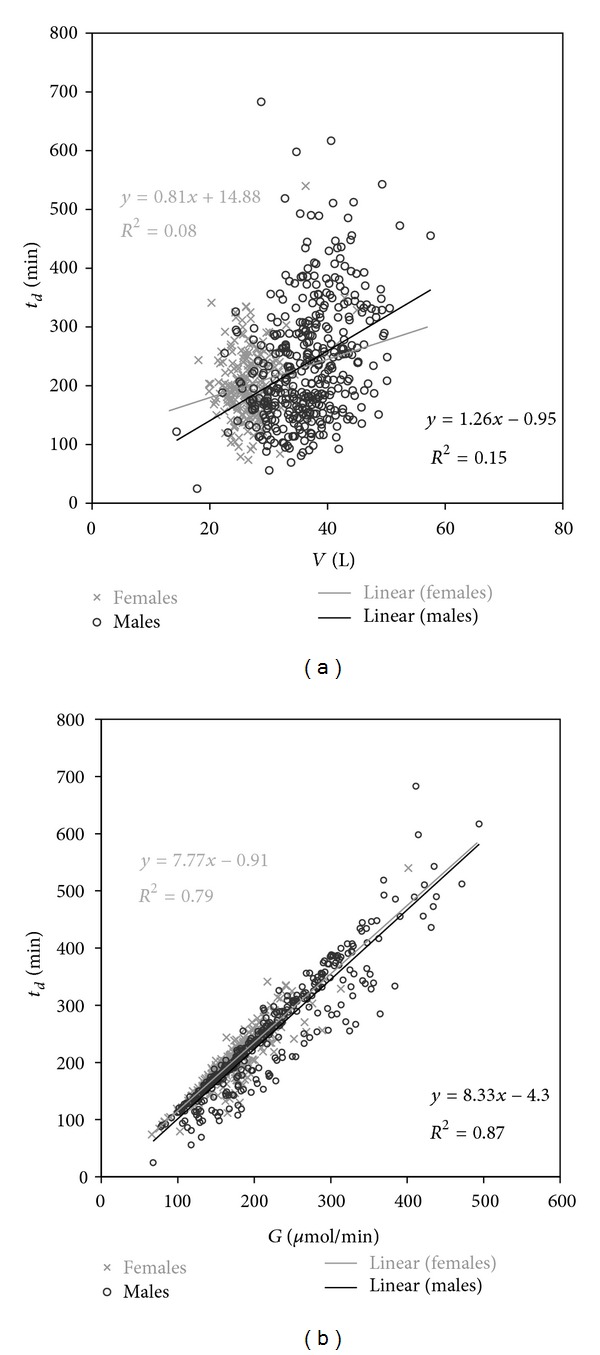
*t*_*d*_ versus *V* and *G* with TAC 17.7 mmol/L in a 3 x/wk schedule; *K*_*d*_ 189 mL/min, *t*_*d*_ adjusted.

The material was dialysed (simulated) three times per week with actual *K*_*r*_ and dialysis clearance *K*_*d*_ 189 mL/min to the mean HEMO-equivalent TAC and PAC by adjusting *t*_*d*_. The required *t*_*d*_ varied widely and had only a weak correlation to *V*, stronger to *G* (Figure3); *t*_*d*_ is determined mainly by *G*. 

To avoid the vicious cycle of underdialysis in patients with low *G*, 164 sessions (26%, TAC target) and 205 sessions (33%, PAC target), where the simulation produced std EKR or std *K*/*V* below that corresponding to *eKt*/*V* 0.9, were dialysed to *eKt*/*V* 0.9 with *K*_*d*_ 189 mL/min (Group 1 in Tables 4 and 5). This resulted in TAC and PAC lower than the mean HEMO-equivalent values. The shortest treatment time was 48 min with std EKR 2.74/wk and 25 min with std *K*/*V* 2.05/wk. For sufficient fluid and toxin removal, longer treatment times may be required.

To avoid extremely long treatment times, 109 (18%) and 142 (23%) (TAC and PAC targets) sessions with simulated *t*_*d*_ > 5 h were changed to a symmetric 5 ∗/wk schedule (Group 3 in Tables 4 and 5). In these groups the mean *G* was over two times that of groups 1. Of course, in many cases it had been possible to increase *K*_*d*_ instead of frequency. 

**Figure 4:**
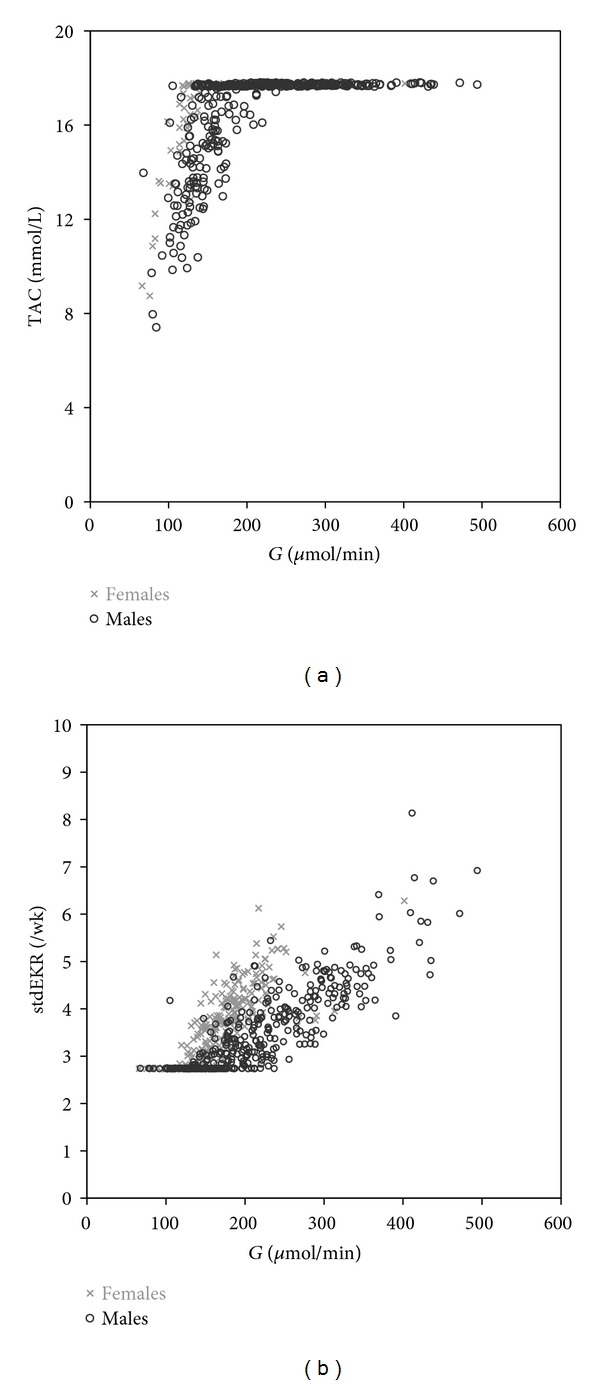
TAC and std EKR versus *G* in dual-targeted dialysis (maximum TAC, minimum std EKR).

With TAC target, 346 sessions (56%), and with PAC target, only 272 sessions (44%) achieved the target in the 3 ∗/wk schedule with reasonable session time (Group 2 in Tables 4 and 5).  Figure 4 and the last column (All) of Tables 4 and 5 describe the whole material dialysed with the dual targets and schedule modifications. 

## 4. Discussion

The present analysis is based on the double-pool variable volume urea kinetic model. The mean kinetic urea distribution volume 33.0 L is 16.5% lower than the mean anthropometric total body water estimate, only 42% of mean body weight (females 40.3, males 43.9), but the patients were overweight (mean body mass index 27.5 kg/m^2^; females 27.0, males 27.9). Daugirdas et al. [25] have observed volume differences of equal magnitude in the HEMO material, where BMI was lower (25.7 kg/m^2^) and kinetically determined volume was 43-44% of body weight.

The correlation between *G* and *V* is weak although they are derived by UKM from the same input variables, permitting mathematical coupling [26, 27]. *G* is quite independent of body size. The relation between *G* and *V* in women is different from that in men. Dialysing to the same *eKt*/*V*, std EKR, or std *K*/*V* results in higher concentrations in women (Table 3). 

nPCR and *G* depend on dietary protein intake (DPI). Low-protein diet may have beneficial effects in uraemia, but rather high protein intake is recommended for dialysis patients. On the average, in this study females seemed to follow the recommendations better than males: nPCR 1.15 versus 1.05 g/day/kg of normal weight, but the variation in nPCR was great (Table 1). This material is too small to conclude whether the difference in nDPI and nPCR between women and men is a universal phenomenon. 

Patient-specific variables *G* and *G*/*V* vary over 7-fold in this unselected material (66–494 *µ*mol/min and 2.0–14.3 *µ*mol/min/L, resp.). Dialysing 3 ∗ 4 h/wk to *eKt*/*V* 1.2 by adjusting dialyser clearance results in a 6.8-fold variation in TAC and 6.5-fold variation in PAC. Dialysing to a constant TAC (17.7 mmol/L, equal to the NCDS lower target and mean HEMO standard dose equivalent TAC) means a 7-fold variation in std EKR (1.15–8.12/wk). 

It is difficult to believe that the huge differences in urea concentrations resulting from *V*-scaled dosing (Table 2) are without significance. The means are probably not the whole truth. The variation is not mere simulation: the mean of the actual predialysis urea concentrations after the longest interval was 23.8, SD 6.5, minimum 7.1, maximum 43.5 mmol/L, and range 6.1-fold. 

Urea concentrations reflect the balance between *G* and *K*. In contrast to the NCDS, in some studies—where DPI, PCR, and *Kt*/*V* were not fully controlled—higher PAC is associated to better outcome [28]. In registry studies the correlation of predialysis urea concentration to mortality is J- or U-shaped [29, 30]. High mortality associated to low urea concentration [31] may be due to malnutrition and wasting caused by comorbidity. High mortality associated to high concentration is due to underdialysis. In cachectic moribund patients, it is easy to achieve low concentrations. 

How should we dialyse patients with unusually low or high dietary protein intake and urea generation rate? Patients with high *G*/*V* have high concentrations when dialysed to a constant *eKt*/*V*, std EKR, or std *K*/*V*. Would they benefit from more intensive treatment? Gotch and Sargent recommend [15, 32] sp *Kt*/*V* 0.9 for patients with low *G* and higher for patients with high *G*, about equal numeric value to nPCR in the 3 ∗/wk schedule. This strategy has seldom been used in outcome studies. In the present study, *eKt*/*V* 0.9 was used as the target in sessions with low *G* (Group 1 in Tables 4 and 5; 26–33% of all sessions). Actually, the simulated dialysis prescription was determined by setting a lower limit for *eKt*/*V*, std EKR, or std *K*/*V* and an upper limit for concentration (Figure 4). The average HEMO-equivalent TAC and PAC (17.7 and 25.4 mmol/L) used here as concentration limits are too low, because they include the low values of sessions with low *G*. Excluding Group 1 gives 19.9 and 28.6 mmol/L, respectively, as averages of TAC and PAC of the remaining 455 sessions. Group 1 is dialysed to *eKt*/*V* 0.9, not to any concentration limit. 

TAC is more stable and results in smaller variation in *Kt* than PAC (SD 17.5 versus 25.3 L). When using PAC as target, high *G* may result in lower TAC than low *G*, but when using TAC as target, high *G* results always in higher PAC than low *G*[33]. TAC had tighter association to outcome than PAC in the NCDS [14]. 

This study reveals great interindividual differences in urea concentrations resulting from using *eKt*/*V*, std EKR, and std *K*/*V* as dialysis dose targets in an unselected population. Perhaps some patients will be underdialysed and some overdialysed with *V*-scaled dosing. If higher normalised DPI, nPCR, *G*/*V*, and urea concentrations in women are a common phenomenon in the dialysis population, it may explain why women did benefit from bigger *V*-scaled dialysis dose in the HEMO study. Using only urea concentration as the target (*G*-scaled dosing) means great modifications to conventional treatment times and schedules, results in unexpected deviations in the elimination of other solutes [34], and endangers the outcomes. The dialysis dose could be determined by setting a lower limit for *V*-scaled clearance (*eKt*/*V* 0.9∗3/wk, std EKR 2.7, or std *K*/*V* 2.1), an upper limit for urea concentration (TAC 20 mmol/L or PAC 30 mmol/L), and a lower limit for time (4 h). 

In the present study, the dialysis treatments were modified afterwards by simulation. This is not possible in real life. Creating a quite accurate prescription is possible by kinetic modelling if we know the patient's *G*, *V*, and *K*_*r*_, but they can vary between sessions, and there are significant error sources in measuring them. 

So long we do not know enough about the metabolism and toxicity of dialysable uraemic solutes, we must search the optimal treatment by trial and error as until now. 

## Figures and Tables

**Table 1:** Modelling sessions of the 35 patients.

Variable	Unit	Mean	SD	Min	Max
Number of sessions		619			
Females	%	37.3			
Age	years	64.7	16.2	16.0	91.6
Height	cm	169	10	150	187
Postdialysis weight	kg	78.9	18.7	43.3	134.5
Body mass index	kg/m²	27.5	5.6	16.0	44.7
Total body water (Watson)	L	39.5	8.1	24.6	61.0
Postdialysis urea distribution volume	L	33.0	7.1	14.4	57.5
Urea generation rate	*μ*mol/min	198	71	**66**	**494**
Normalised protein catabolic rate	g/kg/day	1.09	0.27	**0.48**	**2.34**
Diuresis	L/day	0.25	0.43	0.00	2.30
Renal urea clearance	mL/min	0.77	1.31	0.00	6.79

Note: the most important values are in bold.

**Table 2:** HEMO-equivalent TAC, PAC, stdEKR, and std K/V without RRF (3∗4 h/wk, eKt/V 1.2/session, K_d_ adjusted).

Label	Variable	Unit	Mean	SD	Min	Max
K_d_	Dialyser urea clearance	mL/min	189	37	93	315
*f*_*d*_	Dialysis frequency	/wk	3.00	0.00	3.00	3.00
*t*_*d*_	Dialysis time	min	240	0	240	240
*t*_*dw*_	Weekly dialysis time	h	12.0	0.0	12.0	12.0
wUF	Weekly ultrafiltration volume	L	8.33	2.97	0.55	18.66
TAC	Time-averaged urea concentration	mmol/L	**17.7**	5.2	**5.9**	**40.2**
PAC	Average predialysis urea concentration	mmol/L	**25.4**	7.3	**8.6**	**55.8**
std EKR	std EKR	/wk	**3.44**	0.08	3.23	3.92
std *K*/*V*	std *K*/*V*	/wk	**2.40**	0.07	2.21	2.83

Note: the most important values are in bold.

**Table 3:** Mean TAC and PAC of urea in females and males dialysed to HEMO-equivalent *V*-scaled targets.

Dialysed to	Sex	TAC mmol/L	PAC mmol/L
*eK* *t*/*V*1.2 (without RRF)	Females	18.7	26.8
	Males	17.2	24.6

Std EKR 3.44/wk	Females	18.9	26.3
	Males	17.0	23.8

std K/V 2.40/wk	Females	19.8	27.1
	Males	17.8	24.4

**Table 4:** Dialysis with dual targets: minimum stdEKR and maximum TAC.

Label	Variable	Unit	Group 1		Group 2		Group 3		All	
			Mean	SD	Mean	SD	Mean	SD	Mean	SD
*N*	Number of sessions		164		346		109		619	
*V*	Postdialysis distribution volume	L	35.1	6.1	30.1	6.1	39.0	6.6	33.0	7.1
*G*	Urea generation rate	*μ*mol/min	138	31	190	39	313	57	198	71
nPCR	Normalised protein catabolic rate	g/kg/day	0.77	0.09	1.14	0.16	1.41	0.23	1.09	0.27
K_r_	Renal urea clearance	mL/min	0.49	1.02	0.90	1.37	0.78	1.46	0.77	1.31
*K*_*d*_	Dialyser urea clearance	mL/min	**189**	0	**189**	0	**189**	0	**189**	0
*f*_*d*_	Dialysis frequency	/wk	**3.00**	0.00	**3.00**	0.00	**5.00**	0.00	3.35	0.76
*t*_*d*_	Dialysis time	min	186	37	215	44	207	34	206	42
t_*dw*_	Weekly dialysis time	h	9.3	1.9	10.7	2.2	17.3	2.8	11.5	3.5
wUF	Weekly ultrafiltration volume	L	7.98	2.87	7.81	2.78	10.53	2.70	8.33	2.97
TAC	Time-averaged concentration	mmol/L	14.5	2.2	**17.7**	0.0	**17.7**	0.0	16.8	1.8
PAC	Average predialysis concentration	mmol/L	19.2	2.9	25.1	1.4	23.7	1.0	23.3	3.1
std EKR	std EKR	/wk	**2.74**	0.01	3.64	0.60	4.64	0.86	3.58	0.85
std *K*/*V*	std *K*/*V*	/wk	2.07	0.07	2.56	0.32	3.44	0.50	2.59	0.55

Note: the predefined treatment parameters and target values are in bold.

**Table 5:** Dialysis with dual targets: minimum std *K*/*V* and maximum PAC.

Label	Variable	Unit	Group 1		Group 2		Group 3		All	
			Mean	SD	Mean	SD	Mean	SD	Mean	SD
*N*	Number of sessions		205		272		142		619	
*V*	Postdialysis distribution volume	L	34.7	6.3	30.1	6.1	36.1	7.9	33.0	7.1
*G*	Urea generation rate	*μ*mol/min	143	34	191	40	290	66	198	71
nPCR	Normalised protein catabolic rate	g/kg/day	0.80	0.10	1.14	0.13	1.41	0.21	1.09	0.27
K_r_	Renal urea clearance	mL/min	0.54	1.06	1.01	1.44	0.66	1.33	0.77	1.31
*K*_*d*_	Dialyser urea clearance	mL/min	**189**	0	**189**	0	**189**	0	**189**	0
*f*_*d*_	Dialysis frequency	/wk	**3.00**	0.00	**3.00**	0.00	**5.00**	0.00	3.46	0.84
*t*_*d*_	Dialysis time	min	181	44	208	50	177	42	192	48
t_*dw*_	Weekly dialysis time	h	9.0	2.2	10.4	2.5	14.7	3.5	11.0	3.4
wUF	Weekly ultrafiltration volume	L	7.99	2.87	7.82	2.86	9.80	2.83	8.33	2.97
TAC	Time-averaged concentration	mmol/L	15.5	2.8	18.2	1.2	19.3	0.9	17.6	2.4
PAC	Average predialysis concentration	mmol/L	20.4	3.4	**25.4**	0.1	**25.4**	0.0	23.7	3.1
std EKR	std EKR	/wk	2.70	0.12	3.58	0.65	4.28	0.95	3.45	0.86
std *K*/*V*	std *K*/*V*	/wk	**2.05**	0.00	2.54	0.34	3.23	0.54	2.54	0.55

Note: the predefined treatment parameters and target values are in bold.
